# Spatial multi-omics uncovers an invasive-desmoplastic cellular community with prognostic and therapeutic implications in pancreatic ductal adenocarcinoma

**DOI:** 10.1007/s44307-026-00141-8

**Published:** 2026-10-10

**Authors:** Chang-Hao Liang, Hua Ye, Hui Xu, Chen Fang, Jian-Ping Lian, Yue-Qin Chen, Wen-Tao Wang, Bo He

**Affiliations:** 1 Department of Anesthesiology, and Department of Hepatobiliary, Sun Yat-Sen Memorial Hospital, Sun Yat-Sen University, Guangzhou, 510120 China; 2 MOE Key Laboratory of Gene Function and Regulation, Guangdong Province Key Laboratory of Pharmaceutical Functional Genes, State Key Laboratory of Biocontrol, School of Life Sciences, Sun Yat-Sen University, Guangzhou, 510275 China

**Keywords:** Pancreatic ductal adenocarcinoma, Spatial transcriptomics, Cellular communities, Cancer-associated fibroblasts, Drug repurposing

## Abstract

Pancreatic ductal adenocarcinoma (PDAC) is characterized by a dense desmoplastic stroma and profound spatial heterogeneity, yet the multicellular organizational principles governing its tumor microenvironment remain poorly understood. Here, we performed single-cell RNA sequencing (scRNA-seq; *n* = 9) and spatial transcriptomics (*n* = 4), integrating 61,234 single cells and 30,430 spots to identify an invasive-frontier myofibroblastic cancer-associated fibroblast (IF-myCAF), which is characterized by elevated expression of *ACTA2*, *TAGLN*, *COL11A1*, *THBS2*, *CTHRC1*, and *POSTN*. IF-myCAFs were validated in eight independent PDAC cohorts (159 samples with 67,959 CAFs), showing conserved transcriptional programs and representing a major CAF subpopulation across datasets. Spatial cellular community (CC) analysis identified five recurrent CCs that delineated the multicellular organization of PDAC. Notably, the invasive-desmoplastic CC (ID-CC), which localized to the tumor-stroma interface, was defined by a 5.8-fold enrichment of IF-myCAF; within this community, IF-myCAF colocalized with malignant ductal cells and macrophages. We also found that the ID-CC transcriptional signature was independently associated with worse outcome in TCGA-PAAD. Furthermore, ligand-receptor (LR) analysis of 4,009 IF-myCAF-associated LR pairs identified IF-myCAF as the predominant cell population mediating ECM-related intercellular signaling within ID-CC. Structure-based virtual screening against four IF-myCAF-derived ligand-receptor axes mediating intercellular communication within this community (THBS2-CD47, CTHRC1-FZD8, PPIA-BSG/CD147, MIF-CD74) identified five multi-target drug-repurposing candidates, including conivaptan, lonafarnib, rucaparib, desloratadine, and ketotifen. Collectively, this study first identified a novel CAF subpopulation, IF-myCAF, and established a CC-based organizational scheme for PDAC spatial architecture. Our findings advance our understanding of spatial ecosystem-level stromal heterogeneity in PDAC and suggest targeting IF-myCAF-driven signaling within ID-CC.

**Supplementary Information:**

The online version contains supplementary material available at https://doi.org/10.1007/s44307-026-00141-8.

## Introduction

PDAC remains one of the most lethal malignancies worldwide, with a five-year survival rate below 13% (Siegel et al. 2024). A hallmark of PDAC is its dense desmoplastic stroma, which can constitute up to 70% of tumor volume and is primarily driven by CAFs (Hosein et al. 2020). Seminal scRNA-seq studies have established the existence of major CAF subtypes, including myofibroblastic CAFs (myCAFs), inflammatory CAFs (iCAFs), and antigen-presenting CAFs (apCAFs) (Elyada et al. 2019; Öhlund et al. 2017), and a consensus framework has been proposed to unify CAF nomenclature across cancer types (Sahai et al. 2020). However, a fundamental question remains unresolved: how are these CAF populations spatially organized within intact tumor tissue, and what multicellular neighborhoods do they form to execute their pro-tumorigenic functions?

The functional role of myCAFs in PDAC has been intensely debated. Landmark studies demonstrated that myCAF depletion in genetically engineered mouse models accelerated tumor progression and immunosuppression (Özdemir et al. 2014; Rhim et al. 2014), while others showed that myCAFs promote invasion, metastasis, and therapeutic resistance through matricellular fibrosis and tissue tension (Laklai et al. 2016). Recent work has further complicated this signature by revealing extensive CAF plasticity driven by epigenetic reprogramming (Niu et al. 2024) and MAPK signaling gradients (Veghini et al. 2024), suggesting that CAF functional states are dynamically regulated by microenvironmental cues rather than representing fixed lineages. This contradiction may arise from a critical methodological gap. Single-cell transcriptomics dissociates cells from their native tissue architecture, losing the spatial context essential for linking CAF phenotypes to their precise anatomical localization (Alver et al. 2025). Recently, spatial transcriptomics may provide an opportunity to depict the role of CAFs in PDAC. A recent spatially resolved analysis of PDAC revealed that CAF–malignant cell interactions are extensively remodeled following neoadjuvant therapy, with IL-6 family signaling mediating chemoresistance (Shiau et al. 2024). A spatial multi-omics study integrating transcriptomics, metabolomics, and spatial topology identified three CAF subtypes forming a peri-tumoral-to-distal gradient, with subtype-specific metabolic programs and clinical associations (Zhang et al. 2026a). Thus, elucidating the multilevel role of CAFs in PDAC progression is still an important open question.

Recently, cellular communities (CCs) have been identified by the advanced spatial transcriptomics, which have begun to transform our understanding of tissue architecture in solid tumors. Tumor tissues are organized into the CCs, which spatially defined multicellular ecosystems with conserved cell-type compositions, gene expression programs, and intercellular communication networks across patients (Lin et al. 2026). This CC framework shifts the analytical focus from individual cell types to the multicellular neighborhood as the functional unit of tissue organization (Greenwald et al. 2024; Ma et al. 2026; Ravi et al. 2022). More recently, a pan-cancer analysis of 17 tumor types identified nine conserved spatial ecotypes with distinct cellular compositions, functional programs, and clinical associations (Zhang et al. 2026b), further establishing CC-based tissue organization as a generalizable principle across human cancers. Thus, a systematic CC-based analysis of PDAC may help to define recurrent multicellular communities, characterizing their functional identities, and assessing their clinical relevance.

Here, this study integrated matched scRNA-seq and spatial transcriptomic profiling to establish a CC-based organizational scheme for PDAC spatial architecture. We identified five CCs with distinct cellular compositions and function. Among these, the invasive-desmoplastic CC (ID-CC) is defined by a novel CAF subpopulation, IF-myCAF, which localizes to the tumor invasive frontier in close spatial proximity to malignant epithelial cells and macrophages, representing a functionally specialized, prognostically significant niche associated with PDAC progression. The ID-CC transcriptional signature was associated with poor outcome, positioning it as a potential prognostic marker for PDAC. Furthermore, structure-based drug repurposing targeting ID-CC-specific ligand-receptor complexes identified clinically approved drugs with potential for targeting this pro-tumorigenic niche. This study presented a previously unknown knowledge of spatial ecosystem-level organization of PDAC stromal heterogeneity, and may also provide a foundation for developing spatially therapeutic strategies.

## Materials and methods

### Study cohort and clinical specimens

This study enrolled 5 treatment-naïve patients with pathologically confirmed PDAC who underwent curative surgical resection. Human PDAC tumor and margin tissues were obtained with informed consent from Sun Yat-sen Memorial Hospital and approved by the Hospital’s Protection of Human Subjects Committee (Approval No. SYSKY-2026–585-01). Informed consent was obtained from all subjects. The detailed clinicopathological characteristics of the PDAC patients are summarized in Table S1.

Paired tumor tissue and margin non-tumor tissue (margin tissue) specimens (9 total samples) were collected intraoperatively. The margin tissue was defined as the 1 mm-wide tumor-stroma interface; the tumor tissue was defined as viable tumor ≥ 5 mm from the interface. All specimens were re-validated postoperatively via H&E staining. Tissue samples were processed immediately after resection: one portion for single-cell suspension, and the other embedded in OCT compound, snap-frozen, and stored at −80 °C for spatial transcriptomic assays.

Margin tissue indicates the surgically sampled compartment; The term invasive front describes the region closest to the advancing tumor cells, corresponding to the area where IF-myCAF is enriched in the tumor tissue, as revealed by spatial transcriptomics.

### Single-Cell RNA sequencing

Fresh PDAC tumor tissues were processed into single-cell suspensions via combined mechanical and enzymatic digestion (collagenase IV, hyaluronidase, DNase I). scRNA-seq libraries were prepared using the BaiCreate S2000 Single Cell Transcriptome Kit (Berry Genomics, BaiCreate) following the manufacturer's protocol. Single cells, reverse transcription reagents, and barcoded gel beads were co-encapsulated into nanoliter-scale microdroplets using the BaiCreate Single Cell Controller. Qualified libraries were sequenced on the Illumina NovaSeq 6000 platform (PE150).

### Spatial transcriptomics

Frozen OCT-embedded PDAC tissue blocks were sectioned at 10 μm thickness and mounted onto BaiCreate spatial transcriptome chips (spot diameter 2 μm, each with unique spatial barcodes, providing subcellular resolution exceeding that of the 10 × Visium platform [55 μm spots, 100 μm spacing]). Tissue sections were fixed, stained with H&E, and imaged for histological registration. Tissue permeabilization released intracellular mRNA, which was captured by spatially barcoded oligonucleotide probes. In situ reverse transcription, second-strand synthesis, PCR amplification, and library construction were performed following the manufacturer's protocol. Qualified libraries were sequenced on the Illumina NovaSeq 6000 platform (PE150). Spatial transcriptomic data acquisition and processing followed best-practice guidelines for Visium-like platforms (Moses and Pachter 2022).

### scRNA-seq data processing

Raw sequencing reads were processed using the official BaiCreate single-cell transcriptome analysis pipeline with the GRCh38 human reference genome. Downstream analysis was performed using Seurat v4.4.0 (Hao et al. 2021) in R v4.3.0. Stringent QC filtered cells with < 200 or > 7500 detected genes, or mitochondrial gene ratio > 20%. Gene expression data were normalized using SCTransform. Principal component analysis (PCA) was performed on the top 3000 highly variable genes, and the top 10 PCs were used for Uniform Manifold Approximation and Projection (UMAP) visualization and unsupervised clustering (Louvain algorithm, resolution 0.3–0.5). Cell type annotation was based on canonical marker genes combined with manual curation. Sub-clustering of CAFs, T/NK cells, and epithelial cells was performed by extracting each lineage, re-normalizing with SCTransform, re-running PCA/UMAP, and clustering with optimized resolution (CAFs: 0.4; T/NK: 0.5). CAF subpopulations were annotated based on integrated module scoring: myCAF (*ACTA2*, *TAGLN*, *MYL9*), ECM-CAF (*COL1A1*, *COL3A1*, *SPARC*), complement-secreting CAF (csCAF; *DCN*, *C3*, *CFD*), and a dual ECM–myofibroblastic population, designated IF-myCAF, identified by concordant elevation of ECM-CAF and myCAF module scores (threshold > 0.25 for both). The core regulatory gene set of IF-myCAF was subsequently defined through a multi-dimensional prioritization strategy intersecting four independently derived gene sets. T/NK cell sub-clustering and functional annotation were performed using TCAT (T-CellAnnoTator), a component-based annotation pipeline within the starCAT (CellAnnoTator) framework that resolves T/NK cell subsets and functional states by evaluating consensus Gene Expression Program (cGEP) activity profiles (Kotliar et al. 2025). Malignant epithelial cells were distinguished from normal ductal cells through an integrated strategy combining transcriptomic entropy, PDAC-specific signature scoring, and machine learning-based outlier detection. The gold-standard validation of malignant versus normal epithelial identity was performed using CopyKAT (v1.1.0), which infers large-scale copy number variations (CNVs) from single-cell transcriptomic data (Gao et al. 2021). Cells classified as aneuploid by CopyKAT (harboring chromosome-arm-level CNV events) were designated as malignant epithelial cells, while diploid cells with normal gene expression programs were annotated as normal ductal cells. Immune and stromal cells served as internal diploid references.

### Identification of IF-myCAF Core Regulatory Genes

CAF differentiation trajectories were inferred using Monocle3 (v1.3.0) with the computationally determined transcriptomic root node as the starting state. Genes with dynamic expression patterns along the pseudotime axis were identified by fitting generalized additive models (GAMs) to pseudotime-ordered expression data (*q*-value < 0.05). Trajectory robustness was independently confirmed using Slingshot (v2.6.0) (Street et al. 2018). IF-myCAF core regulatory genes were identified through a four-dimensional prioritization strategy. (1) Trajectory genes: Monocle3 identified genes with significant pseudotemporal variation (*q* < 0.01, Moran's *I* > 0.1, top 700). (2) Differential expression: Genome-wide differential expression analysis was performed comparing IF-myCAF against each other CAF subtype (myCAF, ECM-CAF, csCAF) using the Wilcoxon rank-sum test; genes with |log_2_ fold change|> 1.5 and adjusted *P* < 0.01 in at least one comparison were retained. (3) Highly variable genes: The top 100 highly variable genes within IF-myCAF cells were identified using the variance-stabilizing transformation (VST) method. (4) Co-expression network hub genes: Gene–gene Spearman correlation networks were constructed within IF-myCAF cells; hub genes were defined as the top 10% by connectivity (|*ρ*|> 0.6). Core regulatory genes were defined as those present in at least three of the four gene sets.

### In silico gene knockout analysis

In silico gene knockout was performed using scTenifoldKnk (v0.1.0) on core IF-myCAF genes across two functional modules: ECM Matrix (*COL11A1*, *FN1*, *LAMA1*, *THBS2*) and Oncogenic EMT (*SPP1*, *PPIA*, *MMP11*, *MIF*). For each gene, single-gene knockout was conducted individually (nc-nNet = 3, nc-nCells = 150). Upregulated genes (|*Z*-score|> 2, adjusted *P* < 0.05) were pooled within each module for Gene Ontology (GO) enrichment analysis.

### Cell–cell communication analysis

Intercellular crosstalk was analyzed using CellChat (v2.1.2) with the human ligand-receptor database CellChatDB.human (Jin et al. 2021). Sender-centric and receiver-centric analyses were performed to identify signaling pathways where matrix-rich myCAFs act as the main signal sender or receiver.

### Spatial transcriptomics data processing and CC definition

Raw spatial transcriptomic reads were processed using the official BaiCreate pipeline. QC filtered spots with < 100 detected genes or total UMIs < 500. Cell type deconvolution was performed robust cell type decomposition (RCTD; spacexr v2.2.0) (Cable et al. 2022) in doublet mode, with the annotated scRNA-seq dataset as reference.

Cellular Community (CC) identification: RCTD weight vectors were extracted for each spot. Spatial neighborhood smoothing (*k* = 15 nearest neighbors) was applied to account for local cellular context. Per-sample super-spots (*n* = 200, empirically determined to balance spatial resolution and denoising based on spot density across tissue sections of varying size) were created via k-means clustering of smoothed weight vectors. Cross-sample CCs were identified through multi-method consensus clustering (hierarchical, partitioning around medoids [PAM], and k-means). The number of communities (*k* = 5) was selected using three complementary criteria. First, cross-method agreement, quantified by the mean pairwise adjusted Rand index (ARI) among the three algorithms: the dominant partition (*k* = 2) separated stromal from parenchymal communities (ARI = 0.87), and among fine-grained partitions that resolved stromal heterogeneity (*k* ≥ 3), agreement reached a local optimum at *k* = 5 (ARI = 0.75, range 0.68–0.86; *k* = 3: 0.56; *k* = 4: 0.61; *k* = 6: 0.74; *k* = 7: 0.73; *k* = 8: 0.70), with 92.8% majority-vote agreement. Second, biological interpretability: at *k* = 4, three biologically distinct stromal niches (immune-stromal, fibrotic-stromal, and stromal-remodeling) collapsed into a single mixed community, whereas at *k* = 6, ID-CC was split into two subclusters without new biological identities. Third, per-CC stability under 70% bootstrap resampling (*n* = 30): four of five communities showed Jaccard stability ≥ 0.5 (ID-CC 0.91 ± 0.07; AP-CC 0.98 ± 0.01; FS-CC 0.80 ± 0.11; SR-CC 0.50 ± 0.34), while the smallest community (IS-CC, 1.7% of spots) was consistent across methods but unstable under resampling, as expected for a rare niche. Final CC assignments were taken from the k-means solution, which agreed with the three-method consensus assignment (ARI = 0.90).

Statistical significance of CC spatial coherence was assessed by permutation testing (*n* = 500; toroidal shift of spatial labels). Spatial autocorrelation was quantified using Moran's *I*. CC boundaries were benchmarked against those defined by SPACEL (v1.0) (Xu et al. 2023), confirming the robustness of the consensus clustering approach.

### CC-specific ligand-receptor analysis

CellChat results from scRNA-seq were mapped to CCs by computing CC-specific enrichment scores for each LR pair based on sender and receiver cell-type proportions within each CC. Pathway-level CC activity scores were aggregated across all LR pairs.

### Spatial proximity and gradient analysis

For each spatial sample, nearest neighbor distances between cell-type-enriched spots were computed. Spatial gradient analysis was performed by binning spots along the tumor interior-to-invasive front axis (quantified by malignant ductal proportion (RCTD Ductal fraction × CopyKAT-verified aneuploid fraction)) and computing mean IF-myCAF proportion per bin using GAM smoothing.

### Bulk transcriptome correlation analysis

Gene expression and clinical survival data for 177 PDAC patients were retrieved from the pancreatic adenocarcinoma cohort of The Cancer Genome Atlas (TCGA-PAAD) via TCGAbiolinks. CC signature scores were interpreted strictly as bulk transcriptional correlates reflecting the relative abundance of each CC's constituent cell types, and were used only to test clinical associations of CC-associated transcriptional programs. CC-specific signature scores were calculated using single-sample gene set enrichment analysis (ssGSEA) implemented in the gene set variation analysis (GSVA) package based on the top 50 marker genes per CC. Patients were stratified into high- and low-score groups by median split. Kaplan–Meier survival analysis and multivariate Cox regression (adjusting for stage and age) were performed using the survival and survminer R packages.

### Structure-based drug repurposing

Five ligand-receptor pairs were selected for protein complex structure prediction through parallel evidence channels. CellChat analysis of scRNA-seq data identified 4,009 IF-myCAF-associated LR pairs, of which 2,154 involved IF-myCAF as signal sender (138 unique ligands). MIF-CD74 was prioritized based on its second-highest CellChat communication probability among all IF-myCAF sender pairs (rank 2/138; probability = 0.1094). The top-ranked pair, COL1A1-CD44 (rank 1/138; probability = 0.1103), was deprioritized: CD44 interacts with at least seven collagen ligands in the IF-myCAF secretome (COL1A1, COL1A2, COL4A1, COL4A2, COL6A1, COL6A2, COL6A3; Table S6), resulting in high functional redundancy that limits its specificity as a therapeutic target (Winkler et al. 2020). In contrast, MIF-CD74 represents a less redundant, ligand-specific signaling axis mediating IF-myCAF-to-macrophage crosstalk, with multiple clinical-stage MIF inhibitors available (ISO-1, Ibudilast, Imalumab; Table S10). PPIA-BSG/CD147 (rank 20/138; probability = 0.0323) was prioritized despite its moderate CellChat ranking based on orthogonal functional evidence from in silico single-gene knockout, which identified PPIA as a regulator of core ECM genes (*COL11A1*, *CTHRC1*; see Sect. 2.6), together with cyclophilin inhibitors in Phase III clinical trials (e.g., Alisporivir; Table S10).

In parallel, three IF-myCAF core regulatory genes with literature-supported receptor interactions were nominated independently of CellChat ranking: THBS2-CD47 was nominated through the core-gene channel: THBS2 is a 3/4 core gene and the top-ranked spatially variable gene in ID-CC; among its six CellChat-identified receptors, CD47 was selected over the top-ranked CD36 due to clinical target availability (Magrolimab, Phase III (Sikic et al. 2019)), CTHRC1-FZD8 (*CTHRC1* is a 4/4 core gene and IF-myCAF-defining marker; FZD8 is a Wnt receptor), and COL11A1-ITGA1 (COL11A1 is a 4/4 core gene and IF-myCAF-defining marker). CTHRC1-FZD8 and COL11A1-ITGA1, which are absent from the standard CellChatDB.human ligand-receptor database, were manually curated with literature-supported receptor pairing (CTHRC1 as a Wnt co-receptor binding Frizzled family proteins (Yamamoto et al. 2008); COL11A1 as a collagen XI chain binding α1*β*1 integrin (Zhang et al. 2003)); *THBS2* is present in CellChatDB (rank 22/138) but was nominated through the core-gene channel based on its spatial prominence and clinical target availability. This yielded five LR complexes (MIF-CD74, PPIA-BSG/CD147, THBS2-CD47, CTHRC1-FZD8, COL11A1-ITGA1).

Full-length amino acid sequences were retrieved from UniProt. Protein complex structures were predicted using AlphaFold3-Multimer (AF3) via the AlphaFold Server (Abramson et al. 2024) Protein–protein docking was performed with LightDock using the AF3-predicted monomer structures as input to resolve the ligand-receptor binding interface at near-atomic detail. The binding pocket on the IF-myCAF-derived ligand side of each complex was extracted as the target site for small-molecule screening. COL11A1-ITGA1 was excluded from virtual screening due to its large, shallow binding interface (> 2,000 Å^2^) that lacks a well-defined small-molecule binding pocket, making it unsuitable for conventional structure-based docking. The remaining four targets (MIF, PPIA, THBS2, CTHRC1) possessed structurally resolved, druggable binding pockets and were advanced to virtual screening. A curated library of 3,000 FDA‑approved drugs was screened against these four ligand structures using AutoDock Vina. Cyclosporine A, a known PPIA (CypA) binder, served as a specificity control and was identified among the top-ranked compounds for PPIA, consistent with the established PPIA-binding pharmacology of this drug. Drug-gene interaction data were supplemented via manual curation of DrugBank (Wishart et al. 2018) and published literature.

To further evaluate the specificity and reproducibility of our screening outputs, we performed high-precision re-docking for the five consensus drugs (exhaustiveness = 32; three independent replicates with distinct random seeds), benchmarked them against an empirical null distribution built by docking 99 randomly selected FDA-approved drugs against the same four targets (exhaustiveness = 32; one additional ligand was discarded due to unsuccessful structure preparation), and implemented a decoy-pocket control in which docking was repeated at a non-physiological binding site offset from the predicted pocket. As a decoy-receptor control, the five compounds were also docked to bovine serum albumin (BSA, PDB 3V03). ISO-1, a literature MIF tautomerase inhibitor with micromolar potency, served as an additional weak-binder control.

### External validation in independent PDAC cohorts

Eight independent PDAC scRNA-seq datasets (GSE154778, GSE155698, GSE156405, GSE158356, GSE202051, GSE205013, GSE229413, GSE278694) encompassing 159 samples across multiple platforms (10X Genomics, Drop-seq) were retrieved from Gene Expression Omnibus (GEO). CAFs were extracted from each dataset by standardized marker-based filtering, yielding 67,959 quality-filtered CAFs. Datasets were integrated using Harmony batch correction (30 principal components) followed by unsupervised clustering. Cell-level subtype annotation was performed by consensus scoring of six transcriptional signature modules (myCAF, iCAF, apCAF, csCAF, FAP-CAF, and CD105-CAF), with the IF-myCAF-corresponding population identified using the same dual-module (ECM and myofibroblastic) scoring criterion as in the discovery cohort, revealing 13 transcriptionally distinct CAF subtypes across the merged cohort.

### Statistical analysis

All statistical analyses were performed in R v4.3.0. The two-sided Wilcoxon rank-sum test was used for differential expression and group comparisons; the hypergeometric test for enrichment analysis; Benjamini-Hochberg (BH) correction for multiple comparisons. Spatial correlation was assessed using Spearman's rank correlation. A two-sided adjusted *P* < 0.05 was considered statistically significant.

### Immunofluorescence staining

Formalin-fixed paraffin-embedded (FFPE) PDAC Sects. (4 μm) were deparaffinized, rehydrated, and subjected to heat-induced antigen retrieval in sodium citrate buffer (pH 6.0). The assays were performed as previously described (Chen et al. 2026; Pu et al. 2026). After blocking with 5% BSA-PBS, sections were incubated overnight at 4 °C with primary antibodies against α-SMA (ACTA2; Abcam ab7817, 1:200) and COL11A1 (AWA00725, 1:200). Sections were then incubated with Alexa Fluor-conjugated secondary antibodies and counterstained with DAPI. Fluorescence images were captured at 20 × magnification.

## Results

### Single-cell transcriptomic landscape of paired tumor tissue and margin tissue specimens from PDAC

**Fig. 1:**
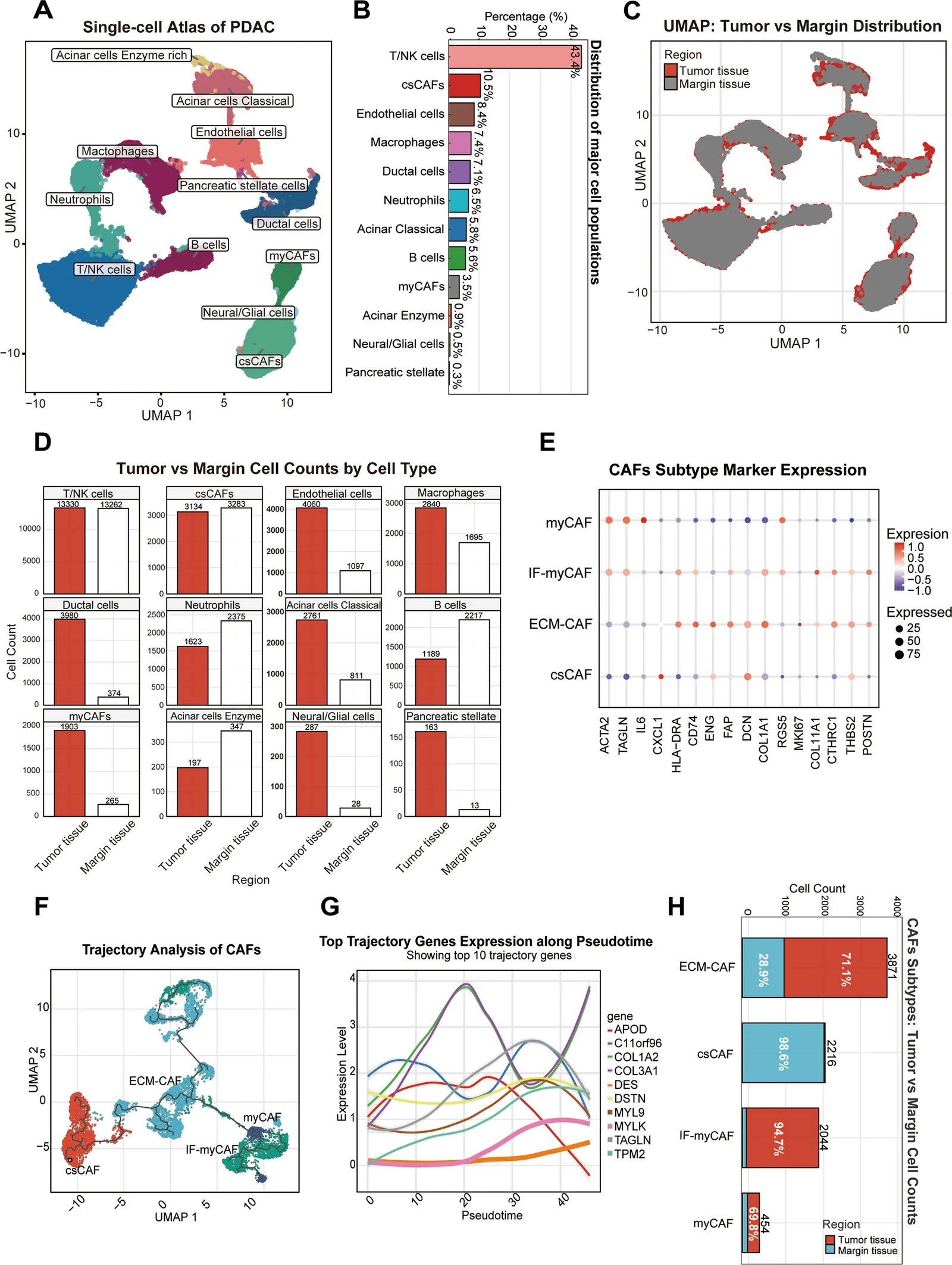
Single-cell transcriptomic landscape of PDAC and identification of CAF subpopulations. **A** UMAP visualization of 61,234 high-quality single cells from paired tumor tissue and margin tissue specimens, colored by 12 major cell lineages. **B** Cellular composition of the tumor microenvironment, with T/NK cells (43.4%) and CAFs (10.5%) as the most abundant components. **C** Integrated UMAP overlay comparing tumor tissue and margin tissue distributions, revealing compartment-specific enrichment of stromal and immune subsets. **D** Quantification of cell-type enrichment across tissue compartments. B cells, neutrophils, and enzyme-rich acinar cells were enriched in the margin tissue; ductal cells, neural/glial cells, and myCAFs were enriched in the tumor tissue. **E** Dotplot of lineage-defining marker genes across four CAF subpopulations. Dot size represents the percentage of cells expressing each gene; color intensity represents the mean expression level. Markers were selected by differential expression analysis (see Methods) combined with literature curation, including myofibroblastic markers (*ACTA2*, *TAGLN*), ECM-remodeling markers (*COL1A1*, *COL11A1*, *THBS2*, *CTHRC1*, *POSTN*), complement-related markers (*DCN*), and others (*RGS5*, *MKI67*). **F** Monocle3 pseudotime trajectory placing IF-myCAF at the terminus of the myofibroblastic differentiation branch. **G** Top 10 pseudotime-associated genes ranked by Moran's *I* along the trajectory. Loess-smoothed curves with 95% confidence intervals. (H) The single-cell transcriptomic landscape of CAF subtypes across paired tumor tissue and margin tissue biopsies. IF-myCAF, ECM-CAF, and myCAF cells were predominantly recovered from tumor tissue specimens (94.7%, 71.1%, and 69.8% respectively), whereas csCAF was almost exclusively recovered from margin tissue specimens (98.6%)

To explore the multicellular organizational principles governing the PDAC microenvironment, paired tumor tissue and margin tissue specimens from 5 treatment-naive PDAC patients were profiled by integrated single-cell RNA-seq and spatial transcriptomics (Table S1). scRNA-seq yielded 61,234 high-quality single cells. UMAP visualization identified 12 major cell lineages, with T/NK cells (43.4%) and CAFs (10.5%) as the most abundant components, alongside macrophages, ductal epithelial cells, endothelial cells, B cells, neutrophils, acinar cells, neural/glial cells, and pancreatic stellate cells (Fig. 1A, B). The cell-type annotations were validated by canonical marker expression profiles (Fig. S1A). Integrated UMAP overlay of tumor tissue and margin tissue compartments revealed profound spatial heterogeneity in cellular composition (Fig. 1C). Cell type-specific abundance analysis demonstrated that B cells, neutrophils, and enzyme-rich acinar cells were enriched in the margin tissue, whereas ductal cells, neural/glial cells, pancreatic stellate cells, endothelial cells, classical acinar cells, and myCAFs were predominantly localized to the tumor tissue (Fig. 1D). Notably, T/NK cells were evenly distributed between the two compartments (13,330 in tumor tissue vs. 13,262 in margin tissue). The coordinated enrichment of distinct stromal, immune, and epithelial subsets within each compartment suggested the existence of higher-order multicellular organizational units in PDAC.

### Subpopulations with distinct transcriptomics and morphological features reveals a novel myofibroblastic CAF

To investigate the functional heterogeneity underlying PDAC progression, three core lineages were subjected to in-depth sub-clustering: ductal epithelial cells, T/NK cells, and CAFs. TCAT sub-clustering of T/NK cells resolved 26 initial clusters into five functionally distinct subsets, including NKT cells, NK cells, Tregs, CD8^+^ memory T cells, and proliferating T cells (Fig. S1B). All subsets met quality thresholds (annotation confidence > 0.5, positive silhouette width; Fig. S1C). Spatial analysis revealed significant compartmentalization: CD8^+^ memory T, NK, and proliferating T cells were enriched in the tumor tissue (62.5% of CD8^+^ memory T cells), while 74% of NKT cells resided in the margin tissue. The CD8^+^ memory T/Treg ratio was significantly reduced in the margin tissue (*P* < 0.001), demonstrating an immunosuppressive microenvironment at the tumor invasive front (Fig. S2A). In addition, cGEP activity mapping revealed convergent effector programming (cytotoxic, CD8^+^ memory) in CD8^+^ memory T and NK cells versus immunosuppressive programming (exhaustion, Treg-specific) in Tregs (Fig. S2B). The corresponding cGEP scores projected onto the UMAP embedding are shown in Fig. S2C.

Ductal epithelial cells were classified using a multi-method strategy combining transcriptome entropy, PDAC-specific signature scoring, machine-learning outlier detection, and CopyKAT CNV analysis (Fig. S3A, B). CopyKAT classified 50.9% of all 4,354 ductal cells as aneuploid (malignant), 37.2% as diploid (normal), and 11.9% as undefined, providing genomic validation independent of transcriptomic classification (Fig. S3C). Moreover, pathway activity scoring also revealed divergent functional programs: normal diploid cells exhibited higher phosphoinositide 3-kinase (PI3K) and transforming growth factor-beta (TGF-β) activity, whereas malignant aneuploid cells selectively activated Hypoxia, nuclear factor kappa-B (NF-κB), WNT, and mitogen-activated protein kinase (MAPK) pathways, which linked to higher tumor survival, metabolic adaptation, and invasion (Fig. S3D).

CAFs constitute an important cell population driving PDAC progression (Hosein et al. 2020). We further investigated this predominant stromal population in the paired tumor tissue and margin tissue specimens. Interestingly, CAFs exhibited a pronounced spatial enrichment difference (Fig. 1D) and were sub-clustered into four functionally distinct subpopulations (Fig. S4A): myCAF, ECM-CAF, csCAF, and a novel myofibroblastic CAF subpopulation (termed IF-myCAF, which was shown by the following spatial transcriptomic analysis to localize to the tumor invasive front). Lineage-defining marker expression and subtype-specific signature scoring validated these annotations (Fig. 1E, S4B). IF-myCAF exhibited a bipartite transcriptional program defined by the concomitant expression of myofibroblastic contractile genes (*ACTA2*, *TAGLN*) and matrisome-associated genes (*COL11A1*, *THBS2*, *CTHRC1*, *POSTN*). This dual signature was not recapitulated in the remaining three CAF subtypes: ECM-CAF expressed the matrisome module but lacked contractile activity, canonical myCAF displayed the contractile module without matrisome activation, and csCAF expressed neither program (Fig. 1E). To further explore the differentiation of these CAF subpopulations, we applied the pseudotime trajectory analysis. The result revealed a branched differentiation structure with ECM-CAF and csCAF at intermediate positions and IF-myCAF at the terminus of the myofibroblastic branch (Fig. 1F). Pseudotime ordering confirmed progressive transcriptional divergence along the myofibroblastic branch, with the top pseudotime-associated genes (ranked by Moran's *I*; Fig. 1G). Among the trajectory-associated genes, the three IF-myCAF-defining markers (COL11A1, THBS2, CTHRC1) were also detected as significant, whose expression was concentrated at the terminal IF-myCAF state, consistent with their terminal differentiation identity (Fig. S4C).

Furthermore, IF-myCAF, ECM-CAF, and myCAF were predominantly recovered from the single-cell transcriptomic of tumor tissue specimens (94.7%, 71.1%, and 69.8%, respectively), whereas csCAF was almost exclusively recovered from margin tissue specimens (98.6%) (Fig. 1H). Pathway analysis further delineated subtype-specific programs: IF-myCAF selectively activated PI3K; ECM-CAF showed elevated WNT activity; csCAF predominantly activated *MYC*, MAPK, and hypoxia; and myCAF displayed elevated p53 and NF-κB signaling (Fig. S4D). Collectively, these scRNA-seq analyses established a comprehensive cellular landscape of the PDAC tumor microenvironment (TME).

To assess the generalizability of IF-myCAF beyond the discovery cohort, eight independent PDAC scRNA-seq datasets encompassing 159 samples and 67,959 quality-filtered CAFs were analyzed (GSE154778, GSE155698, GSE156405, GSE158356, GSE202051, GSE205013, GSE229413, GSE278694). Among 13 identified CAF subtypes, the IF-myCAF-corresponding population was the most prevalent, accounting for 48.4% (32,860/67,959) of all CAFs, followed by ECM-CAF (18.7%), csCAF (10.5%), and canonical myCAF (5.5%) (Fig. S5A-C). Together, we identified a novel myofibroblastic CAF within the tumor mass. This subset may shape PDAC stromal biology and the microenvironment to facilitate PDAC progression.

### Spatial mapping reveals organized tumor architecture and IF-myCAF–malignant cell proximity

**Fig. 2:**
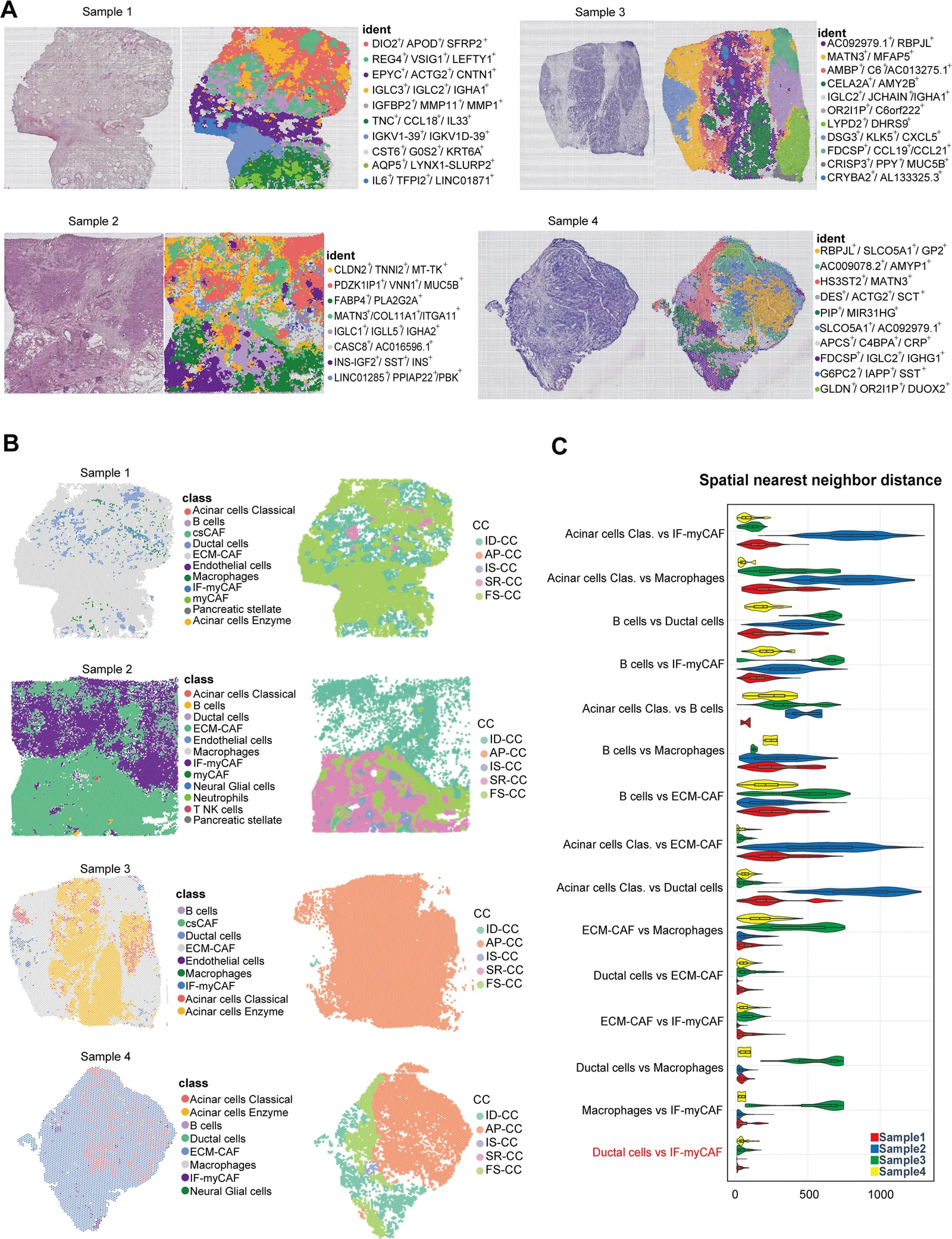
Spatial transcriptomic architecture of PDAC and IF-myCAF–malignant cell proximity. **A** Unsupervised spot clustering projected onto tissue coordinates, recovering the major histological compartments of PDAC without prior histological annotation (molecular zone annotations in Table S2). **B** RCTD deconvolution (left) revealing malignant ductal cells concentrated within tumor nests surrounded by a dense CAF-macrophage stromal mantle; spatial mapping of five recurrent cellular communities (right) across four independent samples. **C** Nearest-neighbor distance analysis identifying IF-myCAF as the cell type in closest physical proximity to malignant ductal cells across multiple independent samples. CC, cellular community; RCTD, robust cell type decomposition

To determine how these populations are physically organized within intact tumor tissue, spatial transcriptomic analysis was next performed. Spatial transcriptomic profiling of matched tissue sections from 4 patients was performed using the BaiCreate S1000 platform, identifying 8–11 transcriptionally distinct spot clusters per sample (Fig. 2A). Projection of cluster identities onto tissue coordinates revealed that transcriptionally similar spots formed spatially contiguous molecular zones (Fig. 2A). Annotation by top differentially expressed genes recovered the major tissue compartments of PDAC without reliance on prior histological labels, including immunoglobulin-defined plasma cell zones, *POSTN*/*COL11A1*-defined stromal zones, *REG4*/*VSIG1*-defined malignant epithelial zones, and acinar (*GP2*, *CELA2A*), islet (*INS*, *SST*), and lymphoid (*FDCSP*, *CCL19*) zones in subsets of samples (Table S2, Fig. 2A).

To further analyze this architecture at cell-type resolution, we next performed the RCTD deconvolution using the scRNA-seq atlas. As shown in Fig. 2B, malignant ductal cells were concentrated within tumor nests, surrounded by a dense CAF-macrophage stromal mantle; acinar cells were restricted to normal parenchyma distal to the tumor; and neural/glial cells localized to discrete regions consistent with neural structures. Notably, IF-myCAF displayed striking spatial patterning relative to malignant ductal epithelium. Spatial gradient analysis and cross-sample meta-analytic generalized additive model (meta-GAM) analysis revealed that IF-myCAF proportion peaked at low malignant ductal abundance (meta-GAM peak at 7.3% malignant ductal proportion, GAM smoothing) and progressively declined toward the tumor center in three of four samples (S1, S3, S4) (Fig. 2B, left). Sample 2 exhibited a quantitatively higher overall IF-myCAF abundance with a right-shifted peak (8.5% malignant ductal), reflecting inter-patient variation in stromal desmoplastic magnitude rather than a qualitatively distinct organizational mode (Fig. S6A). An immunofluorescence assay by co-staining of ACTA2 and COL11A1, the core marker of IF-myCAF, also showed the IF-myCAF at the tumor invasive front (Fig. S6B). These observations reveal a core conserved spatial organization of IF-myCAF at the tumor invasive front, with quantitative inter-patient variation in desmoplastic magnitude. Nearest-neighbor distance analysis confirmed IF-myCAF as the cell type in closest physical proximity to ductal cells across multiple independent samples (ranked closest cell-type pair in both samples tested; Fig. 2C).

### Five cellular communities were identified to define the multicellular organization of PDAC

These findings above indicated that the same cell type may participate in fundamentally different multicellular contexts. To further explore the organized tumor architecture, we moved beyond single-cell-type spatial mapping and developed a cellular community (CC) identification pipeline that employs spatial neighborhood smoothing, super-spot aggregation, and multi-method consensus clustering (hierarchical, PAM, and k-means; at *k* = 5, mean pairwise ARI = 0.75, range 0.68–0.86, majority-vote agreement 92.8%; see Methods).

As a result, five CCs with distinct cell-type compositions were identified. The Invasive-Desmoplastic CC (ID-CC), Acinar-Parenchymal CC (AP-CC), Immune-Stromal CC (IS-CC), and Fibrotic-Stromal CC (FS-CC) were recurrent across multiple tumor-bearing samples, while the Stromal-Remodeling CC (SR-CC) exhibited a more restricted distribution (Table S3; Fig. 2B, right panel). We noted that Invasive-Desmoplastic CC (ID-CC, 6,712 spots) exhibited a 5.8-fold enrichment of IF-myCAF compared to all other CCs, colocalized with malignant ductal cells (3.1-fold enriched), csCAFs (2.8-fold) (Table S3). ID-CC was consistently identified in all four samples (12 spots in the minimally tumor-involved Sample 3; 2,291, 3,775, and 634 spots in Samples 1, 2, and 4, respectively), and the dominant cellular components included ECM-CAF (36.4%), IF-myCAF (15%), and myCAF (11.3%). We have also identified an Acinar-Parenchymal CC (AP-CC, 9,094 spots), which represents the tumor-adjacent pancreatic parenchyma characterized by enzyme-rich acinar cells (55.8%) and ECM-CAF (16.7%). Notably, sample 3 had the least tumor involvement (43.6% acinar cells, < 1% IF-myCAF) and was almost entirely composed of AP-CC, suggesting that AP-CC may be associated with a favorable outcome (Fig. 2B, right). The Immune-Stromal CC (IS-CC, 513 spots) represents a rare, specialized adaptive immune community defined by the highest B cell (13.4%) and T/NK cell (11.5%) enrichment among all CCs, with prominent immunoglobulin, MHC class II, T cell receptor, B cell receptor, and complement signaling programs. The Stromal-Remodeling CC (SR-CC, 4,237 spots) was characterized by ECM-CAF (60.2%), myCAF (11.7%), and pancreatic stellate cells (7.7%) and was predominantly identified in Sample 2. The Fibrotic-Stromal CC (FS-CC, 9,874 spots), the largest stromal CC, was defined by ECM-CAF (58.3%) with the highest macrophage enrichment among all CCs (12.5%) and robust collagen/ECM-remodeling transcriptional programs.

Among the five CCs, ID-CC and FS-CC exhibited the most pronounced tumor-associated features, while AP-CC was characteristic of residual pancreatic parenchyma, IS-CC occupied a specialized adaptive immune niche, and SR-CC occupied an intermediate stromal phenotype. These CCs constructed the multicellular organization of PDAC from pancreatic parenchyma to the tumor center. Spatial autocorrelation analysis confirmed that Moran's *I* was significantly positive across all samples (all *P* < 0.001, permutation test, *n* = 500), validating that CCs represent genuine spatially contiguous multicellular units rather than random cell-type co-occurrence. Of note, ID-CC uniquely combined a tumor-stroma interface spatial localization with a composition enriched for both malignant epithelium and stromal elements, indicating its important role in the development of PDAC.

### Functional specialization distinguishes ID-CC from other cellular communities and its transcriptional program predicts poor outcome in bulk tumor transcriptomes

**Fig. 3:**
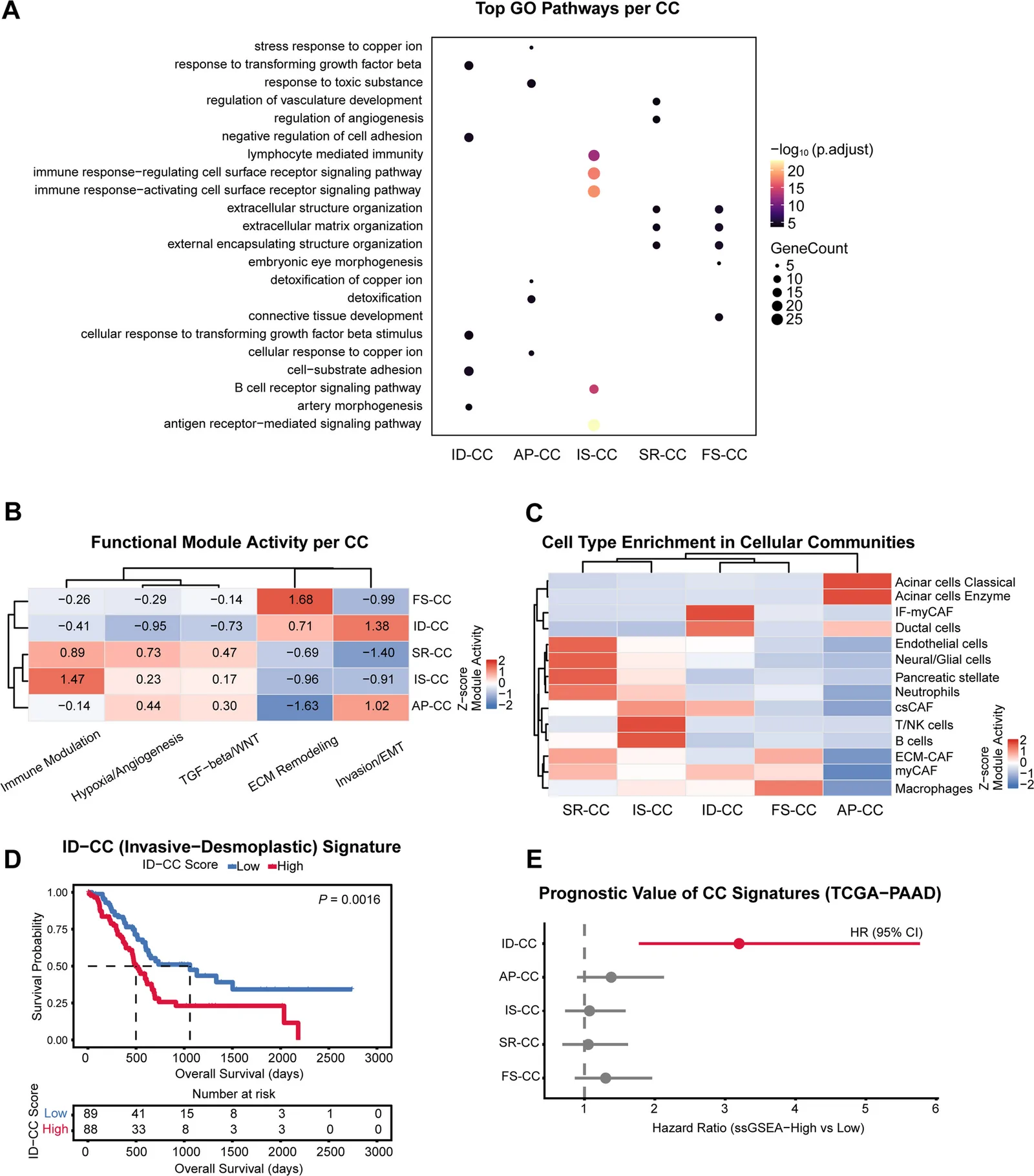
Functional specialization of cellular communities and prognostic significance of the ID-CC signature. **A** GO enrichment of ID-CC-upregulated genes, confirming overrepresentation of cell-substrate adhesion, TGF-β response, ECM organization, and collagen fibril assembly. **B** Functional module scores across five biologically relevant programs (ECM Remodeling, TGF-β/WNT, Hypoxia/Angiogenesis, Immune Modulation, Invasion/EMT). Scores were calculated by GSVA per spot, averaged by CC, and row-wise *Z*-score normalized across CCs for visualization. ID-CC exhibited the highest Invasion/EMT and ECM Remodeling activity among all CCs. The Immune Modulation module was highest in FS-CC (driven by MIF and HLA genes) consistent with its pronounced macrophage enrichment and immune-modulatory stromal character. **C** Cell-type enrichment across CCs. RCTD-derived cell-type proportions were *Z*-score normalized per cell type across CCs (capped at ±2), highlighting CC-specific compositional patterns. **D** Kaplan–Meier overall survival analysis in TCGA-PAAD (*n* = 177). High ID-CC signature scores were significantly associated with worse survival (HR = 1.94, 95% CI 1.28–2.94, log-rank *P* = 0.0016). **E** Forest plot of all five CC signatures. Only ID-CC showed a 95% CI that did not cross 1.0, indicating that adverse prognosis is specifically associated with the transcriptional program of the IF-myCAF-enriched invasive community. GO, Gene Ontology; GSVA, gene set variation analysis; HR, hazard ratio; CI, confidence interval

Having identified the above five CCs with distinct cell-type compositions in paired tumor tissue and margin tissue specimens, we further investigated whether functional specialization differs among them. Differential expression analysis identified 63–150 significantly upregulated genes per CC (FDR < 0.05, log_2_FC > 0.3; Table S4). GO enrichment confirmed overrepresentation of cell-substrate adhesion, TGF-β response, ECM organization, and collagen fibril assembly (Fig. 3A). Functional module scoring across five biologically relevant programs, including ECM Remodeling, TGF-β/WNT, Hypoxia/Angiogenesis, Immune Modulation, and Invasion/EMT, revealed that ID-CC exhibited the highest Invasion/EMT and ECM Remodeling activity among all CCs (Fig. 3B). *Z*-score normalization of cell-type proportions across CCs revealed that ID-CC was uniquely characterized by robust enrichment of IF-myCAF and ductal cells, whereas SR-CC were dominated by stellate cell signatures and AP-CC consisted mainly of acinar cells (Fig. 3C). This result has suggested that stromal CCs exhibited their main signatures and further supported that IF-myCAF enrichment, not ECM-CAF abundance could define the invasive-desmoplastic phenotype of ID-CC.

To further assess clinical relevance of CCs, CC signatures (top 50 upregulated genes each; Table S4) were scored in bulk tumor transcriptomes of the TCGA-PAAD cohort (*n* = 177) using ssGSEA. Kaplan–Meier analysis revealed that high ID-CC scores were significantly associated with worse overall survival (HR = 1.94, 95% CI: 1.28–2.94, log-rank *P* = 0.0016; Fig. 3D). None of the remaining four CC signatures reached prognostic significance (AP-CC: *P* = 0.90; IS-CC: *P* = 0.56; SR-CC: *P* = 0.92; FS-CC: *P* = 0.87; Fig. S6C-F). Among all five CCs, only ID-CC showed a 95% CI that did not cross 1.0 (Fig. 3E), indicating that adverse prognosis is specifically associated with the transcriptional program of the IF-myCAF-enriched invasive community. Multivariate Cox regression confirmed ID-CC as an independent prognostic factor after adjusting for TNM stage and age (HR = 1.92, 95% CI: 1.27–2.92, *P* = 0.002).

### IF-myCAF defines the cellular identity and core regulatory program of ID-CC and dominatingly mediates the ECM-related intercellular signaling

**Fig. 4:**
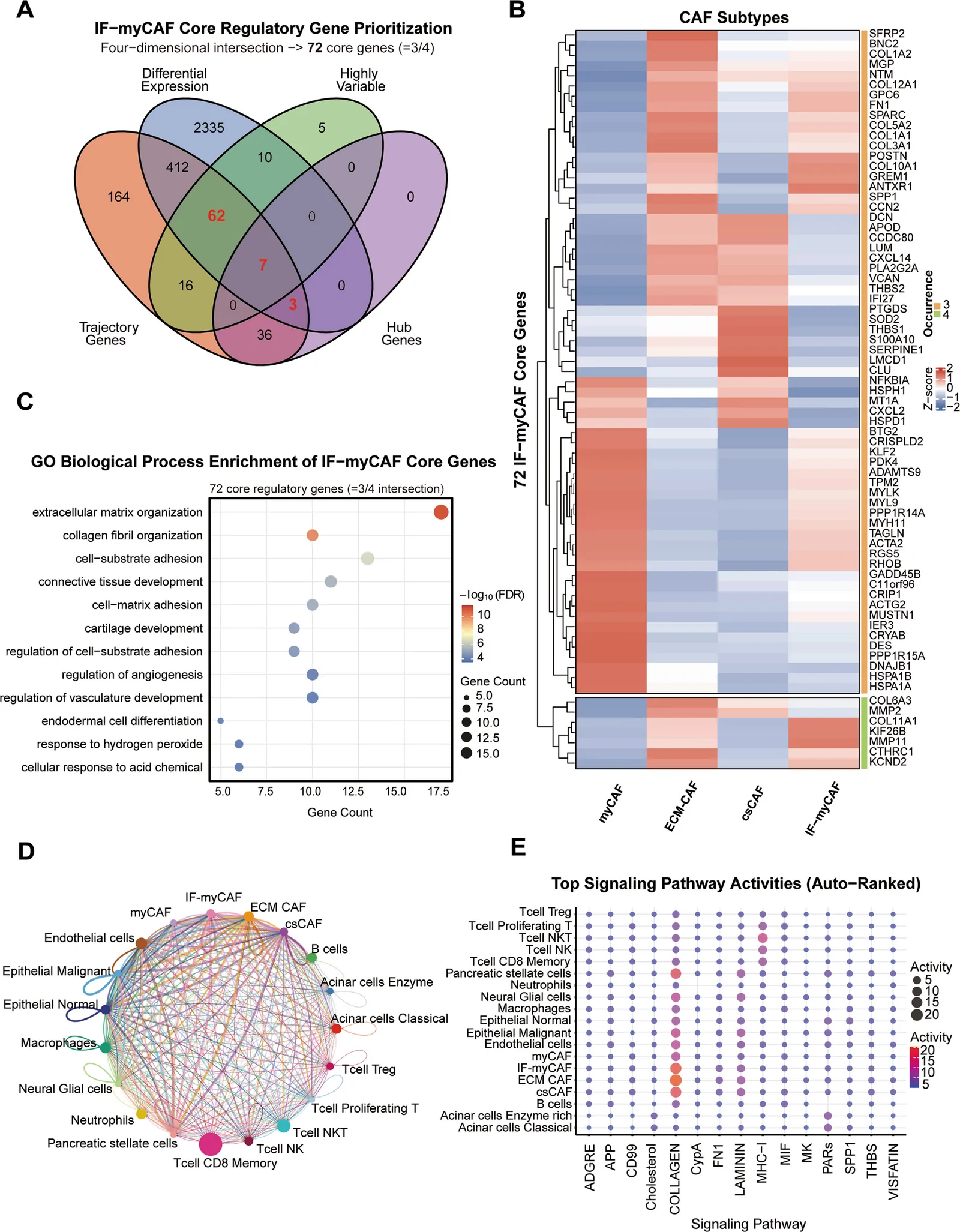
Core regulatory program and signaling landscape of IF-myCAF. **A** Multi-dimensional prioritization strategy integrating CAF differentiation trajectory genes, IF-myCAF-upregulated DEGs, highly variable genes, and co-expression network hub genes, identifying 72 high-confidence IF-myCAF core regulatory genes (Table S5). **B** Heatmap of the 72 core genes across the four CAF subtypes. Expression values are row-wise *Z*-score normalized (capped at ±2), confirming their selective enrichment in IF-myCAF relative to myCAFs, ECM-CAF, and csCAF. Genes are annotated by dimensional occurrence (3/4 vs. 4/4). **C** GO enrichment of the 72 core genes, confirming predominant involvement in ECM organization, collagen fibril assembly, and connective tissue development (FDR < 0.05). **D** CellChat-derived chord diagram of IF-myCAF signaling interactions within ID-CC. Outer segments represent cell types; connecting chords represent aggregated ligand-receptor interactions, with chord width proportional to communication probability. Chords originate from the signal sender and terminate at the receiver, indicating the direction of signaling. **E** CellChat pathway activity heatmap across five cellular communities. Rows represent the top 20 most variable signaling pathways; columns represent CCs. Pathway activity scores were calculated by aggregating communication probabilities of all ligand-receptor pairs within each pathway, weighted by sender and receiver cell-type proportions in each CC. Values are row-wise *Z*-score normalized (capped at ±2). The complete list of 4,009 IF-myCAF-associated LR pairs is provided in Table S6. DEG, differentially expressed gene

To identify the core regulatory genes governing IF-myCAF identity, an integrated multi-dimensional prioritization strategy intersecting four complementary gene sets, including CAF differentiation trajectory genes, IF-myCAF-upregulated DEGs, IF-myCAF highly variable genes, and co-expression network hub genes, identified 72 high-confidence core genes (including the 6 defining marker genes), defined as genes present in at least three of the four gene sets (Table S5, Fig. 4A). Heatmap visualization of the 72 core genes across the four CAF subtypes confirmed their selective enrichment in IF-myCAF relative to myCAFs, ECM-CAF, and csCAF (Fig. 4B). GO enrichment of these 72 genes confirmed predominant involvement in ECM organization, collagen fibril assembly, and connective tissue development (FDR < 0.05; Fig. 4C).

**Fig. 5:**
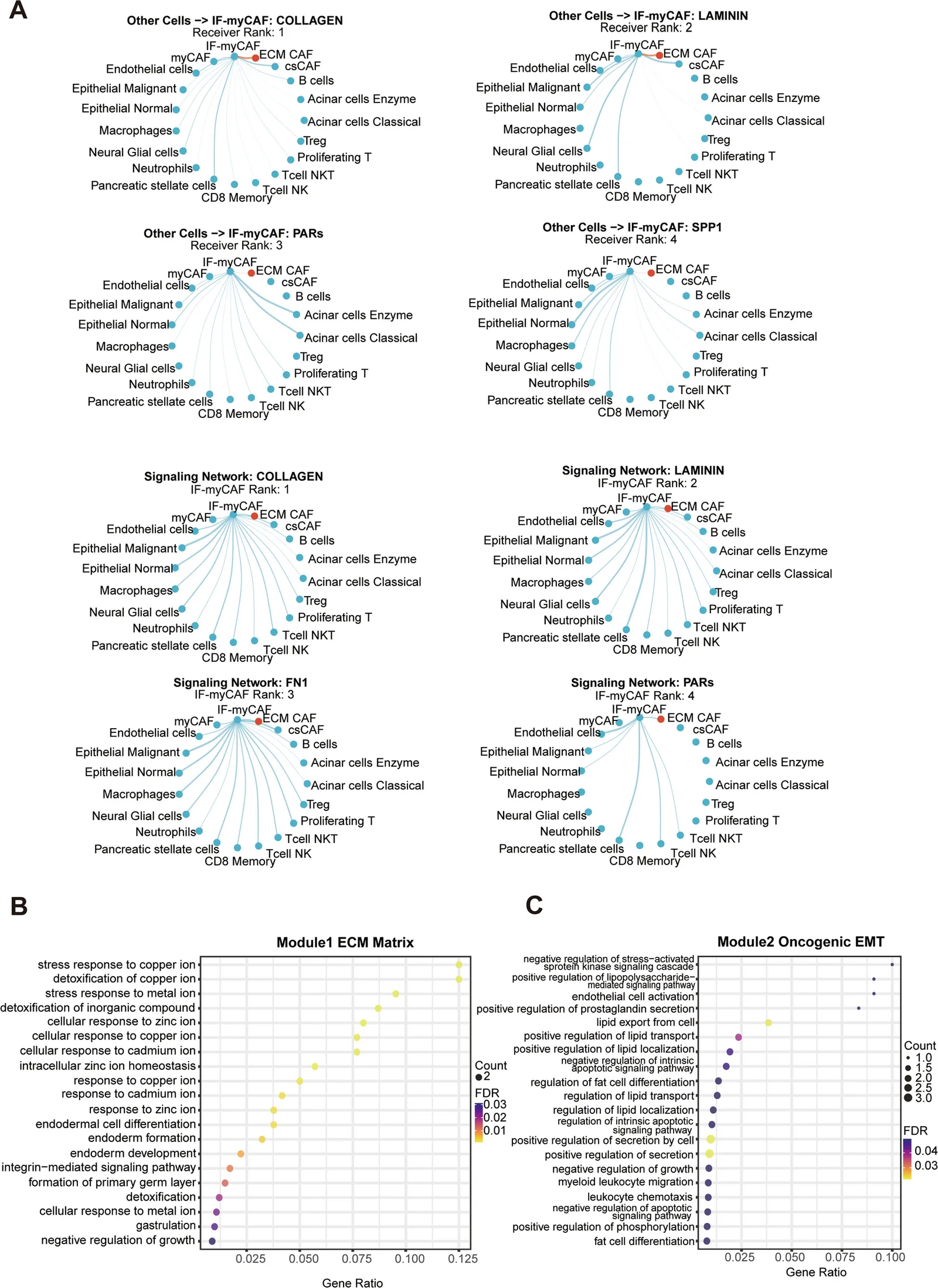
IF-myCAF-centered signaling pathways and in silico functional module perturbation within ID-CC. **A** Top four outgoing and incoming signaling pathways of IF-myCAF within ID-CC. As signal senders, IF-myCAF were dominated by COLLAGEN (aggregate score = 8.65), LAMININ, FN1, and PARs. As receivers, IF-myCAF predominantly sensed COLLAGEN signals (aggregate score = 4.03) from ECM-CAF, suggesting a paracrine collagen-integrin feedforward loop sustaining IF-myCAF activation. **B** GO biological process enrichment of genes upregulated following in silico single-gene knockout of the ECM Matrix module genes (*COL11A1*, *FN1*, *LAMA1*, *THBS2*) using scTenifoldKnk. Upregulated genes (|*Z*-score|> 2, adjusted *P* < 0.05) were pooled across all four knockouts for enrichment analysis. Dot size represents gene count; color represents − log_10_ (FDR). **C** GO biological process enrichment of genes upregulated following in silico single-gene knockout of the Oncogenic EMT module genes (*SPP1*, *PPIA*, *MMP11*, *MIF*), analyzed as in (**B**). Enriched terms include secretion regulation and lipid transport pathways. GO, Gene Ontology; FDR, false discovery rate

To systematically dissect the signaling role of IF-myCAF within ID-CC, CellChat analysis of 30,036 Ligand-receptor (LR) interactions was filtered to those involving IF-myCAF as sender or receiver, identifying 4,009 IF-myCAF-associated LR pairs (Table S6, Fig. 4D, E). As signal senders, IF-myCAF were dominated by the COLLAGEN axis (aggregate score = 8.65), followed by LAMININ, FN1 and THBS (Fig. 5A; Tables S7, S8). As receivers, IF-myCAF predominantly sensed COLLAGEN signals (aggregate score = 4.03), which originated primarily from ECM-CAF, suggesting a paracrine feedforward loop sustaining IF-myCAF activation. PARs and SPP1 constituted additional incoming signaling axes (Fig. 5A; Table S7). In addition, spatial analysis within ID-CC revealed that IF-myCAF co-localized with macrophages (Spearman *r* = 0.31), consistent with their inferred COLLAGEN-CD44 and FN1-CD44 signaling. IF-myCAF and ductal cells, while in closest physical proximity by nearest-neighbor distance (Fig. 2C), occupied adjacent but distinct spatial domains (Spearman *r* = − 0.18), consistent with juxtacrine signaling across the tumor-stroma interface (Table S9).

To complement the signaling analysis, in silico single-gene knockout was performed on core IF-myCAF genes within the ECM Matrix (*COL11A1*, *FN1*, *LAMA1*, *THBS2*) and oncogenic EMT (*SPP1*, *PPIA*, *MMP11*, *MIF*) modules using scTenifoldKnk. Module-level GO enrichment of upregulated genes revealed that ECM Matrix module perturbation activated stress response and metal ion detoxification programs, while Oncogenic EMT module perturbation enriched secretion regulation and lipid transport pathways (Fig. 5B, C). Together with the CellChat findings, these results establish IF-myCAF as the dominant ECM signaling hub of ID-CC, with their LR interactions functionally coupled to both matrix production and immune modulation.

### Structure-based drug repurposing identifies candidates targeting IF-myCAF-driven signaling

**Fig. 6:**
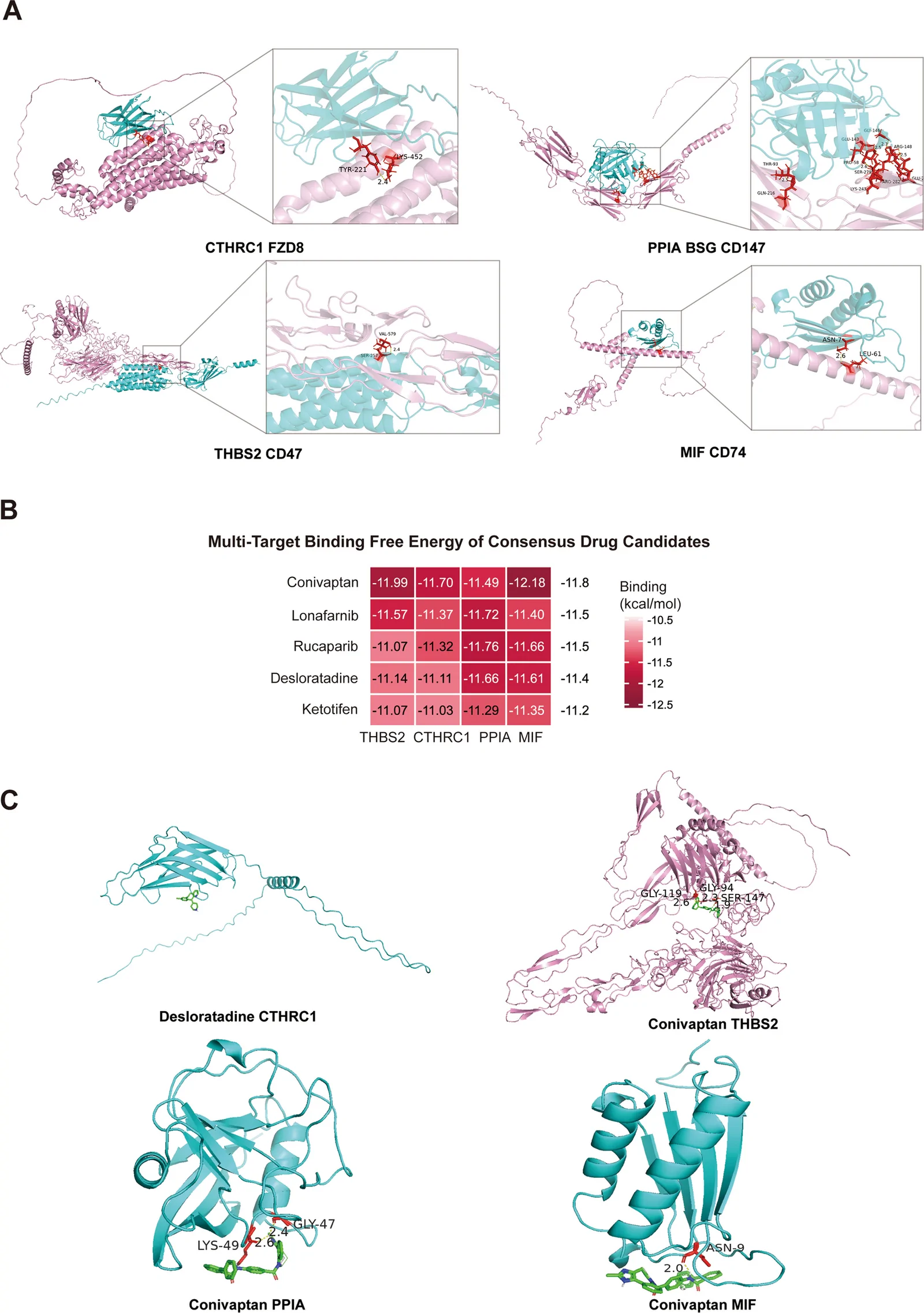
Structure-based drug repurposing targeting IF-myCAF-derived LR complexes. **A** AlphaFold3-Multimer predicted structures and LightDock protein–protein docking of four IF-myCAF-derived LR complexes (THBS2-CD47, CTHRC1-FZD8, PPIA-BSG/CD147, MIF-CD74), showing interface residue details with hydrogen-bond networks and hydrophobic contacts defining druggable binding pockets. COL11A1-ITGA1 was excluded owing to a large, shallow interface (> 2,000 Å^2^). **B** Top five multi-target drug candidates from virtual screening of 3,000 FDA-approved drugs, ranked by mean binding affinity across all four targets (high-precision re-docking means; Table S11): conivaptan (−11.84 kcal/mol), lonafarnib (−11.52), rucaparib (−11.45), desloratadine (−11.38), and ketotifen (−11.18). **C** Representative binding poses of the five consensus drugs showing hydrophobic and hydrogen-bond interaction networks within the predicted target pockets. LR, ligand-receptor

We next explored the potential structure-based drug that may target IF-myCAF-driven signaling. These four LR pairs were prioritized through parallel evidence channels: CellChat communication probability ranking (MIF-CD74, rank 2/138; COL1A1-CD44 deprioritized due to CD44 ligand promiscuity), in silico knockout validation (PPIA-BSG/CD147), and core-gene status with literature-curated receptor pairing (THBS2-CD47, CTHRC1-FZD8); COL11A1-ITGA1 was excluded after AlphaFold3 prediction due to a non-druggable interface (> 2,000 Å^2^) (Fig. S7, Methods 2.12). AlphaFold3-Multimer structure prediction and LightDock protein–protein docking subsequently generated the complex conformations of the four advanced LR pairs: THBS2-CD47, CTHRC1-FZD8, PPIA-BSG/CD147, and MIF-CD74 (Fig. 6A). Interface residue analysis revealed specific hydrogen-bond networks and hydrophobic contacts defining the binding pockets of each pair, providing actionable active-site templates for competitive small-molecule targeting (Fig. 6A). We further investigated the potential structure-based drug by virtual screening of 3,000 FDA-approved drugs against these four structures. As a result, we identified five drugs with consensus mean binding affinities ≤ − 11.0 kcal/mol across all four targets: conivaptan (−11.84 kcal/mol), lonafarnib (−11.52), rucaparib (−11.45), desloratadine (−11.38), and ketotifen (−11.18) (Fig. 6B). Binding pose inspection revealed that each drug engaged its target through distinct hydrophobic and hydrogen-bond interaction networks within the predicted pocket (Fig. 6C).

Because the five consensus drugs are pharmacologically unrelated and were selected against four unrelated targets, we performed computational orthogonal validation of the screening results (Table S11). High-precision re-docking reproduced the consensus ranking (mean affinities −11.84 to −11.18 kcal/mol), and the five drugs ranked in the top 1.1% of an empirical null distribution of 99 randomly sampled FDA-approved drugs re-docked with the same exhaustiveness for every target (empirical *P* ≤ 0.0101). Offset-pocket docking yielded markedly weaker affinities for the compact single-domain targets (PPIA: − 6.52 vs −11.58; MIF: − 6.58 vs −11.64 kcal/mol; *P* < 0.01) but was non-informative for the large multidomain targets (THBS2, CTHRC1). Positive controls behaved as expected (Cyclosporine A showed its best affinity at PPIA; the weak MIF inhibitor ISO-1 scored weakly at all four targets). The five drugs also engaged serum albumin's hydrophobic cavities with high affinity (BSA: −12.1 to −13.6 kcal/mol), consistent with their lipophilic pharmacokinetics. Together with literature-curated evidence (Table S10), these analyses show that the candidates reproducibly outperform the null distribution at all four pockets; their broad hydrophobic binding affinity indicates that the docking-based nominations remain computational hypotheses requiring functional validation, and the translational interpretation is accordingly framed as therapeutic implications.

We have also identified 14 additional drug-gene interactions involving ID-CC hub genes (Table S10), including five FDA-approved or clinically advanced agents, notably Losartan and Pirfenidone (*COL1A1* synthesis), Cyclosporine A (*PPIA/CypA*), Magrolimab (anti-CD47, Phase III), and Metuximab (anti-CD147, approved in China). Together, virtual screening and literature-based repurposing provide a set of potential structure-based drugs that may target IF-myCAF-driven signaling and could reveal a new avenue for developing strategies to treat PDAC.

## Discussion

By integrating matched scRNA-seq and spatial transcriptomics of paired tumor tissue and margin tissue specimens, this study resolved the multicellular organization of PDAC into five recurrent cellular communities and identified a dual contractile-matrix CAF state, IF-myCAF, that defines an invasive-desmoplastic community (ID-CC) whose transcriptional signature carries independent prognostic significance in bulk tumor transcriptomes. The signaling axes through which IF-myCAF organizes this niche harbor pharmacologically tractable interfaces, nominating repurposing candidates that warrant experimental follow-up.

The identification of IF-myCAF may help reconcile the long-standing controversy over the tumor-promoting versus tumor-restraining functions of myCAFs (Özdemir et al. 2014; Rhim et al. 2014). IF-myCAF co-expresses the contractile program of canonical myCAFs and the matrisome program of ECM-CAFs, occupies the terminus of the myofibroblastic differentiation branch, and concentrates at the invasive front where malignant epithelial nests confront the desmoplastic mantle. This positioning argues that CAF function cannot be read from cell identity alone: the same broad stromal lineage may assume opposing roles depending on its multicellular context, as an ECM-CAF embedded in benign parenchyma participates in tissue maintenance whereas its counterpart engaged in ID-CC sustains invasive-desmoplastic signaling. Consistent with the growing recognition that CAF states are plastic, microenvironment-driven phenotypes rather than fixed lineages (Niu et al. 2024; Veghini et al. 2024; Zhang et al. 2025), and with spatial analyses showing that CAF subtype topology rather than bulk abundance tracks immune contexture (Zhang et al. 2026a). CAF consensus framework emphasizes that spatial context, cellular neighborhood, and tissue-specific function must be integrated into CAF classification schemes (Sahai et al. 2020). The CC-based organizational scheme may directly address this call by defining the multicellular context in which specific CAF populations operate, providing a path toward reconciling contradictory functional studies, at least in PDAC. Future therapeutic strategies should target not CAF subtypes in isolation, but rather the specific multicellular communities they define and the microenvironmental signals that sustain them.

At the ecosystem level, the CC-based organization observed here parallels recent findings in glioblastoma (Lin et al. 2026) and pan-cancer spatial analyses (Zhang et al. 2026b; Kang et al. 2024), supporting the emerging view that recurrent multicellular communities constitute the functional units of tumor architecture. PDAC nevertheless exhibits distinctive features: the extreme ECM dominance of its invasive community, the strong prognostic signal of the ID-CC signature relative to the other four community signatures, and the absence of the neuroglial synaptic component characteristic of GBM (Greenwald et al. 2024; Ravi et al. 2022). Notably, the ID-CC signature was the only CC signature independently associated with survival in TCGA-PAAD, suggesting that prognostic information resides in the specific composition of the invasive-desmoplastic niche rather than in stromal abundance per se — a distinction that conventional H&E-based tumor-stroma ratio assessment cannot capture. As a bulk-level correlate of this composition, the ID-CC signature, anchored by THBS2, which has been established as a circulating biomarker for PDAC detection (Kim et al. 2017) and whose stromal expression has been linked to immune exclusion (Liao et al. 2022), may complement histopathological grading for patient stratification. These conclusions are, however, derived from spatial profiling of four patients; the conserved IF-myCAF program across eight external cohorts (159 samples, 67,959 CAFs) supports generalizability at the cell-state level, but the community architecture itself should be validated in larger independent spatial cohorts.

Within ID-CC, IF-myCAF functions as a bidirectional ECM signaling hub: it dominates COLLAGEN output while sensing collagen signals from ECM-CAF, a paracrine loop that may sustain matrix remodeling within the community, with CD44 serving as a multifunctional receptor in collagen- and hyaluronan-rich stroma (Misra et al. 2015). The enrichment of MIF-CD74, PPIA-CD147, THBS2-CD47, and CTHRC1-FZD8 among IF-myCAF-derived axes couples stromal matrix production to immune modulation, as both MIF and CypA (PPIA) are secreted pro-inflammatory mediators implicated in macrophage polarization and chemoresistance (Nigro et al. 2013), and CAF-derived paracrine factors have been shown to support tumor cells while suppressing anti-tumor immunity (Francescone et al. 2021). This provides a mechanistic rationale for the immunosuppressive character of the margin tissue, reflected in the reduced CD8 + memory T/Treg ratio. Structure-based screening — a repurposing strategy with established promise in oncology (Pushpakom et al. 2019) — against these axes, using AlphaFold3-predicted complex structures as atomic-level templates (Jumper et al. 2021), nominated five multi-target repurposing candidates; the five compounds also bound serum albumin's hydrophobic cavities with comparable affinity, consistent with their lipophilic pharmacokinetics. Their docking-based nomination therefore remains a hypothesis until validated in functional assays, and their clinical relevance is best framed as therapeutic implications to be tested experimentally.

Collectively, this study first identified a novel CAF subpopulation, IF-myCAF, and established a CC-based spatial organizational scheme for PDAC. IF-myCAF is characterized by high-expression of *ACTA2*, *TAGLN*, *COL11A1*, *THBS2*, *POSTN*, and *CTHRC1*, representing a major CAF subpopulation across PDAC datasets. Notably, IF-myCAF defines an invasive-desmoplastic community, termed ID-CC, which represents a functionally specialized niche associated with PDAC progression and whose transcriptional signature is independently associated with poor outcome. We further found that IF-myCAF-derived signaling axes within ID-CC may be targetable by existing approved drugs. Collectively, these findings advance the understanding of PDAC stromal heterogeneity from single-cell cataloguing to spatial ecosystem-level organization and provide a foundation for spatially informed therapeutic strategies.

## Supplementary Information

Supplementary Material 1: Fig. S1. Cell-type annotation and T/NK cell sub-clustering. Fig. S2. T/NK cell spatial compartmentalization and cGEP activity. Fig. S3. Ductal epithelial cell classification and CopyKAT CNV validation. Fig. S4. CAF subpopulation functional annotation. Fig. S5. External validation of IF-myCAF conservation across eight independent PDAC cohorts. Fig. S6. Spatial gradient and prognostic analysis of PDAC cellular communities. Fig. S7. Multi-criteria selection of ligand-receptor complexes for structure-based drug repurposing.

Supplementary Material 2: Table S1. Clinical characteristics of the 5 PDAC patients.

Supplementary Material 3: Table S2. Molecular zone annotations and marker genes for each spot cluster.

Supplementary Material 4: Table S3. Cellular composition of five CCs with enrichment fold-changes.

Supplementary Material 5: Table S4. Differentially expressed genes per CC and top 50 CC-specific signature genes.

Supplementary Material 6: Table S5. Seventy-two high-confidence core regulatory genes of IF-myCAF.

Supplementary Material 7: Table S6. Complete list of 4,009 IF-myCAF-associated ligand-receptor pairs.

Supplementary Material 8: Table S7. Outgoing and incoming signaling pathway scores of IF-myCAF within ID-CC, ranked by CellChat aggregate communication score.

Supplementary Material 9: Table S8. Source cell-type contributions to COLLAGEN signals received by IF-myCAF within ID-CC.

Supplementary Material 10: Table S9. Spearman spatial colocalization coefficients between cell-type pairs within ID-CC.

Supplementary Material 11: Table S10. Literature-curated drug-gene interactions involving ID-CC hub genes.

Supplementary Material 12: Table S11. Orthogonal validation of structure-based virtual screening.

## Data Availability

The single-cell RNA-seq and spatial transcriptomic data generated in this study have been deposited in NCBI under accession number PRJNA1481364. The external validation datasets analyzed in this study are publicly available from GEO under accessions GSE154778, GSE155698, GSE156405, GSE158356, GSE202051, GSE205013, GSE229413, and GSE278694.
