# Supplementary material for: Spatial multi-omics uncovers an invasive-desmoplastic cellular community with prognostic and therapeutic implications in pancreatic ductal adenocarcinoma
Source: Adv Biotechnol (Singap). 2026 Oct 10;4(4):39. doi: 10.1007/s44307-026-00141-8 (PMC13653914; doi:10.1007/s44307-026-00141-8)
Supplement: Supplementary file 1 [file 44307_2026_141_MOESM1_ESM.docx]

**Supplementary Materials**

**Supplementary Figures**

**Fig. S1 Cell-type annotation and T/NK cell sub-clustering.** (A) Validation of 12 major cell lineage annotations using canonical marker gene expression. Dot size represents the percentage of cells expressing each marker; color intensity represents the mean expression level. Marker genes were selected from established single-cell atlases and the CellMarker database. (B) TCAT sub-clustering of T/NK cells resolving 26 initial clusters into five functionally distinct subsets: NKT cells, NK cells, Tregs, CD8+ memory T cells, and proliferating T cells. (C) Quality assessment of T/NK cell subset annotations by TCAT. Annotation confidence scores (bars, left y-axis) and mean silhouette width (points, right y-axis) are shown for each of the 26 initial clusters. The dashed line indicates the confidence threshold (>0.5); all retained subsets exhibited positive silhouette widths, confirming robust cluster separation.

**Fig. S2 T/NK cell spatial compartmentalization and cGEP activity.** (A) Quantitative comparison of T/NK subset abundance between the tumor tissue and margin tissue. CD8+ memory T, NK, and proliferating T cells were enriched in the tumor tissue, while NKT cells were enriched in the margin tissue. The CD8+ memory T/Treg ratio was significantly reduced in the margin tissue (*P* < 0.001). (B) Heatmap of consensus Gene Expression Program (cGEP) activity scores across five T/NK cell subsets. cGEP scores were calculated by non-negative least squares (NNLS) deconvolution using the TCAT reference matrix, averaged per subset, and row-wise *Z*-score normalized. The top 20 most variable cGEPs are shown, revealing convergent effector programming (Cytotoxic, CD8-EM) in CD8+ memory T and NK cells versus immunosuppressive programming (Exhaustion, Treg) in Tregs. (C) UMAP visualization of representative cGEP activity scores across individual T/NK cells. Color intensity represents the cGEP activity score (NNLS-derived, plasma colormap). Key functional programs shown include Cytotoxic, Exhaustion, CellCycle-S, CellCycle-G2M, Treg, CD4-Naive, CD8-Naive, CD8-EM, ISG, and TH1-Like.

**Fig. S3 Ductal epithelial cell classification and CopyKAT CNV validation.** (A) Multi-method classification of malignant versus normal ductal epithelial cells. Left: UMAP feature plots of four classification metrics — integrated malignant score (combining entropy, PDAC signature, and outlier scores), transcriptome entropy, core epithelial score, and PDAC-specific signature score. Right: violin plots showing the distribution of each metric across malignant and normal ductal populations (Wilcoxon test *P* values shown). (B) UMAP visualization of ductal cells classified into normal (diploid) and malignant (aneuploid) populations. (C) CopyKAT CNV classification of 4,354 ductal cells. Stacked bar chart showing 50.9% aneuploid (malignant), 37.2% diploid (normal), and 11.9% undefined. Normal reference pool: 43,688 immune and stromal cells (T/NK, B cell, Endothelial, Macrophage, Neutrophil). (D) Pathway activity scores comparing malignant (aneuploid) and normal (diploid) ductal epithelial cells. Pathway activities were calculated using PROGENy or, where unavailable, GSVA with manually curated pathway gene sets. Boxplots show the distribution of activity scores for nine cancer-relevant pathways (PI3K, MAPK, TGF-β, WNT, NF-κB, MYC, Hypoxia, p53, JAK-STAT) across malignant (red) and normal (blue) ductal populations. Asterisks indicate significance: *P* < 0.05, *P* < 0.01, *P* < 0.001 (two-sided Wilcoxon test).

**Fig. S4 CAF subpopulation functional annotation.** (A) UMAP visualization of four functionally distinct CAF subpopulations: myCAF, ECM-CAF, csCAF, and invasive-front matrix-rich myofibroblastic CAF (IF-myCAF). (B) Subtype-specific signature module scores confirming transcriptional coherence of the four CAF subpopulations (*n* = 8,585 cells). For each of the seven modules shown, the Kruskal-Wallis test across subtypes was significant (all *P* < 1 × 10^−134^), and IF-myCAF was compared with each of the other three subtypes using Dunn's post hoc tests (Benjamini-Hochberg-corrected); all 21 comparisons were significant (*P* < 1.3 × 10^−6^), annotated as in the figure (***). (C) Pseudotime expression profiles of IF-myCAF-associated matrisome genes. Loess-smoothed expression of COL11A1, CTHRC1, and THBS2 along the Monocle3 pseudotime trajectory. All three genes exhibit stepwise upregulation toward the IF-myCAF terminus (Bin 10), with COL11A1 showing the most pronounced terminal restriction (near-zero expression in Bins 1–8, peak at Bin 10). Shaded regions represent 95% confidence intervals. (D) Pathway activity heatmap across four CAF subpopulations. Pathway activities were calculated using PROGENy with nine cancer-relevant pathways (PI3K, MAPK, TGF-β, WNT, NF-κB, MYC, Hypoxia, p53, JAK-STAT). Values represent median pathway activity per CAF subtype, row-wise *Z*-score normalized (capped at ±2). Asterisks indicate FDR < 0.05 (Kruskal-Wallis test with Benjamini-Hochberg correction). IF-myCAF selectively activated PI3K; ECM-CAF showed elevated WNT activity; csCAF predominantly activated MYC, MAPK, and Hypoxia; and myCAF displayed elevated p53 and NF-κB signaling.

**Fig. S5 External validation of IF-myCAF conservation across eight independent PDAC cohorts.** (A) UMAP visualization of 67,959 quality-filtered CAFs from 159 samples across eight external PDAC scRNA-seq datasets (GSE154778, GSE155698, GSE156405, GSE158356, GSE202051, GSE205013, GSE229413, GSE278694), colored by annotated CAF subtype. (B) UMAP clustering of external validation CAFs into 25 initial subclusters prior to subtype annotation. (C) Bar chart of CAF subtype composition in the merged external cohort, with the IF-myCAF-corresponding population (*COL11A1*+ *THBS2*+ *CTHRC1*+) as the most prevalent CAF subtype (48.4%, 32,860 / 67,959).

**Fig. S6 Spatial gradient and prognostic analysis of PDAC cellular communities.** (A) Cross-sample spatial gradient analysis of IF-myCAF distribution relative to malignant ductal epithelium. GAM smoothing curves are overlaid for all four PDAC spatial transcriptomic samples (S1–S4, colored lines), with a cross-sample meta-GAM consensus curve (thick grey line, 95% confidence band) revealing a conserved unimodal pattern: IF-myCAF peaks at low malignant ductal abundance (meta-GAM peak at 7.3% malignant ductal proportion) and declines toward the tumor center. Sample 2 (blue) exhibits higher overall IF-myCAF abundance with a right-shifted peak (8.5%), reflecting quantitative inter-patient variation in desmoplastic magnitude rather than a qualitatively distinct organizational mode. The x-axis represents malignant ductal proportion (RCTD Ductal fraction multiplied by the CopyKAT-verified aneuploid fraction [scalar = 0.509]) as a quantitative proxy for proximity to the malignant compartment. (B) Representative immunofluorescence staining of Sample 1. Section was co-stained by COL11A1 (green), ACTA2 (red), and DAPI (blue), revealing IF-myCAF cells at the tumor invasive front. 5× or 20× magnification was shown. Scale bar: 50 µm. (C-F) Kaplan-Meier overall survival analysis of the four non-ID-CC signatures in TCGA-PAAD (*n* = 177). Patients were stratified by median split of ssGSEA scores. None of the four signatures reached prognostic significance: AP-CC (C, *P* = 0.90), IS-CC (D, *P* = 0.56), SR-CC (E, *P* = 0.92), FS-CC (F, *P* = 0.87).

**Fig. S7 Multi-criteria selection of ligand-receptor complexes for structure-based drug repurposing.** (A) Evidence matrix for five LR complex candidates across four selection criteria: CellChat evidence, IF-myCAF core gene membership, literature-supported drug target, and structural druggability. Dot size and color intensity reflect evidence strength: dark blue, strong/direct evidence; light blue, moderate or indirect evidence; grey, absent. *COL11A1*-*ITGA1* was excluded at the structural assessment step (red cross). (B) Two-path selection flow diagram. Left: CellChat-based discovery. Among 4,009 IF-myCAF-associated LR pairs, 2,154 involved IF-myCAF as signal sender (138 unique ligands). *MIF*-*CD74* (rank 2/138) and *PPIA*-*BSG*/*CD147* (rank 20/138, additionally validated by in silico knockout) were prioritized based on communication probability. Right: Core gene-based discovery. *THBS2*-*CD47*, *CTHRC1*-*FZD8*, and *COL11A1*-*ITGA1* were nominated from the 72 IF-myCAF core regulatory genes with literature-supported receptor pairing. *CTHRC1* and *COL11A1* are absent from the CellChatDB.human ligand-receptor database and were manually added. Both paths converge at five candidate complexes. Structural assessment of the AlphaFold3-predicted binding interfaces identified four complexes with well-defined druggable pockets. *COL11A1*-*ITGA1* was excluded owing to a large, shallow binding interface (>2,000 Å²).
